# Serious infections in people with systemic sclerosis: a national US study

**DOI:** 10.1186/s13075-020-02216-w

**Published:** 2020-06-29

**Authors:** Jasvinder A. Singh, John D. Cleveland

**Affiliations:** 1 Medicine Service, Birmingham Veterans Affairs (VA) Medical Center, 700 19th St S, Birmingham, AL 35233 USA; 2 Department of Medicine at School of Medicine, University of Alabama at Birmingham, 510 20th Street South, Birmingham, AL 35294-0022 USA; 3 Division of Epidemiology at School of Public Health, University of Alabama at Birmingham, 1720 Second Ave South, Birmingham, AL 35294-0022 USA

**Keywords:** Serious infections, Systemic sclerosis, Incidence, Time trends, Outcomes, Opportunistic infections, Skin and soft tissue infections, Urinary tract infection, Pneumonia, Sepsis

## Abstract

**Objective:**

To study incidence, time trends, and outcomes of serious infections in systemic sclerosis (SSc).

**Methods:**

We used the 1998–2016 US National Inpatient Sample data. We examined the epidemiology, time trends, and outcomes of five serious infections (opportunistic infections (OI), skin and soft tissue infections (SSTI), urinary tract infection (UTI), pneumonia, and sepsis/bacteremia) in hospitalized people with SSc. We performed multivariable-adjusted logistic regression analyses to analyze independent association of factors with healthcare utilization (hospital charges, length of hospital stay, discharge to non-home setting) and in-hospital mortality.

**Results:**

There were 49,904,955 hospitalizations with serious infections in people without SSc and 61,615 in those with SSc. During 1998–2016, the most common serious infections in SSc were pneumonia (45%), sepsis (32%), SSTI (19%), UTI (3%), and OI (3%). In 2013–2014, sepsis surpassed pneumonia as the most common serious infection; by 2015–2016, sepsis was 1.8 times more common than pneumonia. Over the study period, hospital charges increased, while length of hospital stay and in-hospital mortality decreased, overall and for each serious infection. Multivariable-adjusted analyses showed that sepsis, age ≥ 80 years, and Deyo-Charlson score ≥ 2 were associated with significantly higher odds of healthcare utilization and in-hospital mortality, and Medicare or Medicaid insurance payer, Northeast location, urban teaching or non-teaching hospital, and medium or large hospital bed size with significantly higher odds of healthcare utilization.

**Conclusions:**

Outcomes in people with SSc hospitalized with serious infections have improved over time, except higher hospital charges. Identification of factors associated with higher healthcare utilization and in-hospital mortality allows for developing interventions to improve outcomes.

## Key messages

1. Sepsis and pneumonia are the most common serious infections in systemic sclerosis.
2. Over a 19-year study period, the length of hospital stay and in-hospital mortality decreased, overall and for each serious infection, in people with systemic sclerosis.
3. A diagnosis of sepsis (relative to other serious infections), age ≥ 80 years, and Deyo-Charlson score ≥ 2 were associated with significantly higher odds of healthcare utilization and in-hospital mortality in systemic sclerosis patients hospitalized with a serious infection.

## Background

Systemic sclerosis (SSc) is a multisystem autoimmune disease, associated with high morbidity and mortality [1] and frequent hospitalizations [2]. In an analysis of national US data from 2002 to 2003, the most common reasons for hospitalizations in patients with SSc were diseases of the circulatory (22%), gastrointestinal (13%), musculoskeletal (12%), and respiratory system (11.5%) [3]. Respiratory infection (8%) was the third leading cause of mortality (principal diagnoses) in hospitalized SSc patients and ranked higher than heart failure [3]. In a study of 116 patients with SSc examined over 14 years with a median follow-up of 2 years, of the 31 people who died, 13 (11%) died of infections [4]. In a systematic review of infections in connective tissue diseases, most studies were limited to lupus, and only one study included people with SSc [5]. Therefore, while infection has a significant contribution to mortality in people with SSc [3, 4], few studies have examined the epidemiology of hospitalized infections and their outcomes in SSc [5]. Therefore, our study objective was to examine the epidemiology, time trends, healthcare utilization, and mortality of serious infection hospitalizations in SSc in a national US cohort.

## Methods

### Data source and study cohort selection

Our study cohort included five, common serious infection hospitalizations in people with SSc in the US NIS 1998–2016 sample. The NIS is a 20% stratified sample of discharge records from all participating community hospitals from all participating states [6]. The NIS is the largest publicly available, de-identified all-payer inpatient health care database in the USA. It has been used for epidemiological studies of hospitalization, mortality, and costs, since it represents all hospitalizations in the USA. The Institutional Review Board at the University of Alabama at Birmingham (UAB) approved this study.

We identified five types of serious infections based on the presence of International Classification of Diseases, Ninth Revision, Clinical Modification (ICD-9-CM) codes in the primary diagnosis position for hospitalization: (1) opportunistic infections (OI; 010.xx –018.xx, 031.xx, 078.5, 075.xx, 053.xx, 112.4, 112.5, 112.81, 112.83, 130.xx, 136.3, 117.5, 027.0, 039.xx, 117.3, 114.xx, 115.xx, 116.0); (2) skin and soft tissue infections (SSTI; 040.0, 569.61, 681.xx, 682.xx, 785.4, 728.86, and 035.xx); (3) urinary tract infection (UTI; 590.xx); (4) pneumonia (003.22, 481.0, 513.0, 480.xx, 482.xx, 483.xx, 485.xx, 486.xx); and (5) sepsis/bacteremia (038.xx and 790.7), as previously [7, 8]. These diagnostic codes have been shown to be valid in administrative datasets, with positive predictive values of 70 to 100% in people with rheumatoid arthritis [9]. We also used the ICD-10-CM codes for infections for the 2015–2016 data due to a coding system change to ICD-10-CM in 2015 in the USA. (Appendix 1). Composite infection was defined as any of the serious infection occurring as primary diagnosis for hospitalization. SSc was identified based on the presence of an International Classification of Diseases, ninth or tenth revision, clinical modification (ICD-9-CM or ICD-10-CM) code 710.1 or M34 (includes progressive systemic sclerosis, CREST syndrome, scleroderma, acrosclerosis, but excludes circumscribed scleroderma) in a non-primary position during the index hospitalization. A previous study showed sensitivity of 81% and specificity of 95% using a diagnostic code approach for SSc [10].

### Covariates and outcomes

We adjusted each regression model for covariates/confounders, including age, sex, race, serious infection type (OI, SSTI, UTI, pneumonia, sepsis [reference]), median household income, the insurance payer, hospital characteristics (region, location/teaching status, bed size), and Deyo-Charlson comorbidity index, a validated measure of medical comorbidity that includes 17 comorbidities (myocardial infarction, congestive heart failure, cerebrovascular disease, dementia, renal disease, liver disease, chronic pulmonary disease, diabetes, etc.), based on the presence of ICD-9-CM codes at index admission [11], with higher score indicating more comorbidity load. Deyo-Charlson index was categorized as none, one, or two or above, as previously [12–14].

We examined healthcare utilization and in-hospital mortality, and the details are as follows: (1) health care utilization: total hospital charges above the median for each calendar year; the length of hospital stay above the median of 3 days; and discharge to non-home settings (rehabilitation or an inpatient facility); and (2) in-hospital mortality.

### Statistical analyses

We followed the survey analysis procedures that account for the weights, clusters, and strata as defined in NIS, including the modified weights with the change in sampling in 2012. We compared the summary statistics using chi-square or Student’s *t* test, as appropriate. Rates were calculated per 100,000 NIS claims. We analyzed time trends in rate of each serious infection using Cochran Armitage test. We performed multivariable-adjusted logistic regression analyses for each study outcome, adjusting for all covariates listed in the section above. Odds ratios (OR) and 95% confidence intervals (CI) were calculated. We used SAS 9.3 (Cary, NC) for all analyses. We considered a *p* value < 0.05 to be statistically significant, which corresponds to a 95% CI that excludes unity.

## Results

### Characteristics and outcomes of people with vs. without SSc hospitalized with serious infection

There were 49,904,955 hospitalizations with serious infections in people without SSc and 61,615 in those with SSc. The average age of patients with SSc with a serious infection was 61.4 years (median of 61.7 years; Appendix 2), similar to all SSc hospitalizations.

Compared to patients admitted with serious infection without SSc, people with SSc were younger (median age, 65 vs. 62 years), were more likely to be female (52% vs. 84%), or have Deyo-Charlson score of 2 or more (42% vs. 64%; Appendix 2).

Compared to patients hospitalized with serious infection without SSc, people with SSc had higher median hospital charges ($16,832 vs. $22,105) and a longer median hospital stay (3.7 vs. 4.4 days); were more likely to have hospital stay > 3 days (59% vs. 66%), hospital charges above the median (75% vs. 79%), or to be discharged home (74% vs. 83%); and had a higher in-hospital mortality (6.2% vs. 9%) (Appendix 2).

### Serious infection type in SSc: characteristics and outcomes

Over the study period 1998–2016, the most common serious infections in SSc were pneumonia (45%) and sepsis (32%), followed by SSTI (19%), UTI (3%), and OI (3%; Appendix 3). SSc patients hospitalized with pneumonia or sepsis were 5 years older than those admitted with OI (Appendix 3).

The median length of hospital stay over the study period was the highest for OI hospitalizations at 6.1 days and the lowest for UTI at 2.8 days (Appendix 3). Serious infections led to above median length of hospital stay in 61–79% of discharges except for UTI, with 45% of the people. Median hospital charges were highest for hospitalizations with sepsis at $38,118 and lowest for UTI, $13,646 (Appendix 3).

### Time trends in serious infection hospitalization in SSc and associated healthcare utilization and mortality

**Fig. 1:**
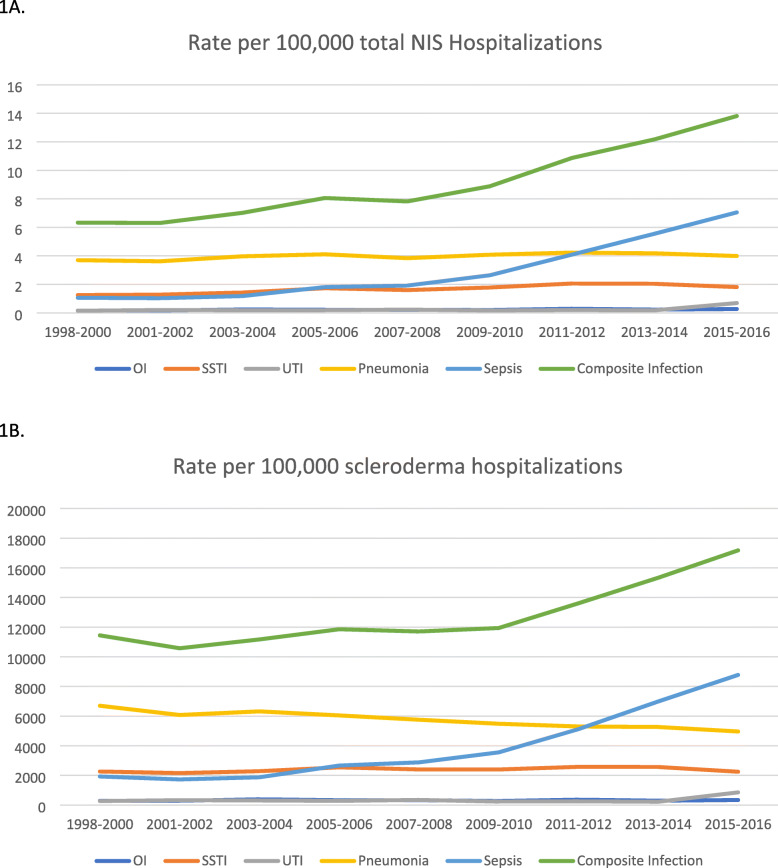
Rate of hospitalized infection in people with scleroderma per 100,000 total NIS claims (**a**) and per 100,000 overall scleroderma claims (**b**)

We noted a significant increase in the overall frequency of sepsis, and possibly OI, SSTI, and UTI (Appendix 4). In comparison, rates in the general population increased for sepsis and SSTI, possibly for OI and decreased for pneumonia (Appendix 5). Rates per 100,000 NIS claims increased for sepsis, OI, SSTI, and UTI (except pneumonia) over time in people with SSc (Appendix 6; Fig. 1). Using all scleroderma claims as the denominator, findings were replicated except that we noted a decreasing rate of hospitalized pneumonia over time relative to all scleroderma hospitalizations (Appendix 6)**.**

By 2013–2014, sepsis surpassed pneumonia as the most common serious infection in people with SSc, and by 2015–2016, sepsis accounted for 1.8 times as many hospitalized serious infections as pneumonia (Appendix 6; Fig. 1).

We found a 3.6-fold increase in the overall mean hospital charges for composite serious infection in SSc patients from $23,152 in 1998–2000 to $87,095 in 2015–2016 (Appendix 7). On the other hand, the mean hospital stay decreased from 4.6 days in 1998–2000 to 4.2 days in 2015–2016. The reduction was the greatest for OI, from 9.5 to 5.8 days. In-hospital mortality also decreased from 10.3% for composite serious infection to 7.8%, respectively. The largest reductions were for OI, 21.2 to 7.7%; sepsis, 22.5 to 13.2%; and pneumonia, 9.8 to 2.8% (Appendix 7). Trends in mean hospital stay and total hospital charges across all study periods are shown in Appendix 8.

### Predictors of healthcare utilization and mortality in SSc admitted with serious infection

Multivariable-adjusted analyses showed that compared to sepsis, other infections were significantly associated with lower healthcare utilization and mortality; age ≥ 80 years and Deyo-Charlson score ≥ 2 were also significantly associated with higher healthcare utilization and mortality (Table 1). Medicare or Medicaid insurance payer, Northeast location, urban teaching or non-teaching status, and medium or large hospital bed size were associated with higher odds of healthcare utilization only (Table 1).

**Table 1:** Multivariable-adjusted correlates of healthcare utilization and mortality for serious infections in scleroderma

	Hospital charges > median	Discharge to care facility	Length of hospital stay > 3 days	In-hospital mortality
	Adjusted odds ratio (95% CI)			
**Age category**				
< 50 years	**Ref**	**Ref**	**Ref**	**Ref**
50 to < 65 years	1.06 (0.95, 1.19)	**1.39 (1.19, 1.62)**	1.10 (0.99, 1.22)	**1.54 (1.24, 1.90)**
65–79 years	0.96 (0.85, 1.09)	**2.01 (1.70, 2.38)**	1.12 (0.99, 1.27)	**2.08 (1.65, 2.63)**
≥ 80 years	**0.79 (0.68, 0.93)**	**4.17 (3.43, 5.08)**	**1.23 (1.05, 1.44)**	**3.23 (2.46, 4.24)**
**Sex**				
Male	**Ref**	**Ref**	**Ref**	**Ref**
Female	0.99 (0.89, 1.11)	0.98 (0.85, 1.11)	1.03 (0.93, 1.14)	0.93 (0.78, 1.10)
**Race/ethnicity**				
White	**Ref**	**Ref**	**Ref**	**Ref**
Black	1.10 (0.96, 1.26)	1.13 (0.96, 1.33)	1.07 (0.94, 1.22)	1.15 (0.93, 1.43)
Hispanic	**1.20 (1.03, 1.39)**	**0.79 (0.65, 0.96)**	0.95 (0.83, 1.09)	1.15 (0.92, 1.44)
Other/missing	1.09 (0.98, 1.21)	0.98 (0.86, 1.12)	**1.14 (1.03, 1.27)**	1.20 (1.00, 1.44)
**Deyo-Charlson score**				
0	Not est	Not est	Not est	Not est
1	**Ref**	**Ref**	**Ref**	**Ref**
≥ 2	**1.54 (1.42, 1.68)**	**1.37 (1.23, 1.53)**	**1.44 (1.33, 1.57)**	**1.49 (1.28, 1.74)**
**Income category**				
0–25th percentile	**0.81 (0.71, 0.91)**	0.94 (0.81, 1.09)	1.00 (0.89, 1.13)	0.82 (0.68, 1.00)
25–50th percentile	**0.83 (0.74, 0.93)**	0.90 (0.78, 1.04)	1.01 (0.91, 1.13)	1.03 (0.86, 1.24)
50–75th percentile	**0.85 (0.76, 0.96)**	0.96 (0.84, 1.10)	1.00 (0.90, 1.12)	0.95 (0.80, 1.13)
75–100th percentile	**Ref**	**Ref**	**Ref**	**Ref**
**Primary infection diagnosis**				
Sepsis	**Ref**	**Ref**	**Ref**	**Ref**
OI	0.87 (0.66, 1.15)	**0.38 (0.27, 0.54)**	**1.48 (1.11, 1.97)**	**0.46 (0.30, 0.70)**
SSTI	**0.42 (0.37, 0.47)**	**0.34 (0.29, 0.40)**	**0.65 (0.58, 0.73)**	**0.06 (0.04, 0.09)**
UTI	**0.28 (0.23, 0.36)**	**0.34 (0.25, 0.46)**	**0.32 (0.26, 0.41)**	**0.02 (0.00, 0.11)**
Pneumonia	**0.64 (0.58, 0.70)**	**0.38 (0.34, 0.43)**	**0.72 (0.66, 0.79)**	**0.29 (0.25, 0.34)**
**Insurance payer**				
Medicare	**1.16 (1.05, 1.30)**	**1.76 (1.52, 2.03)**	**1.21 (1.10, 1.35)**	**0.80 (0.67, 0.95)**
Medicaid	**1.22 (1.05, 1.42)**	**1.48 (1.20, 1.82)**	1.12 (0.97, 1.29)	0.90 (0.68, 1.18)
Other	0.92 (0.69, 1.21)	1.29 (0.86, 1.93)	0.93 (0.71, 1.22)	1.46 (0.95, 2.25)
Private	**Ref**	**Ref**	**Ref**	**Ref**
Self	1.28 (0.93, 1.77)	0.79 (0.46, 1.38)	1.06 (0.78, 1.43)	1.08 (0.60, 1.94)
**Hospital region**				
Northeast	**Ref**	**Ref**	**Ref**	**Ref**
Midwest	**0.75 (0.66, 0.85)**	1.00 (0.86, 1.16)	**0.83 (0.73, 0.94)**	**0.66 (0.53, 0.81)**
South	0.92 (0.82, 1.03)	**0.83 (0.72, 0.95)**	0.95 (0.85, 1.07)	0.86 (0.71, 1.03)
West	0.89 (0.78, 1.02)	0.85 (0.73, 1.00)	**0.68 (0.60, 0.77)**	0.83 (0.68, 1.01)
**Hospital location/teaching**				
Rural	**Ref**	**Ref**	**Ref**	**Ref**
Urban non-teaching	**2.57 (2.26, 2.92)**	**0.74 (0.63, 0.87)**	**1.38 (1.21, 1.57)**	1.18 (0.93, 1.49)
Urban teaching	**2.25 (1.99, 2.54)**	**0.63 (0.54, 0.74)**	**1.31 (1.15, 1.48)**	1.15 (0.91, 1.45)
**Hospital bed size**				
Small	**Ref**	**Ref**	**Ref**	**Ref**
Medium	**1.46 (1.29, 1.65)**	1.05 (0.90, 1.23)	**1.20 (1.06, 1.36)**	1.23 (0.99, 1.55)
Large	**2.10 (1.89, 2.35)**	1.01 (0.88, 1.16)	**1.36 (1.22, 1.52)**	**1.35 (1.10, 1.66)**

*CI* confidence interval, *Ref* reference category; Odds ratio in **bold font** indicate that they were statistically significant with a *p*-value <0.05, i.e., the 95% confidence interval excludes 1.0

## Discussion

In this national study of people with SSc hospitalized with serious infection, we found that compared to patients hospitalized with serious infection without SSc, people with SSc had higher healthcare utilization and in-hospital mortality. In people with SSc, hospital charges and the length of hospital stay were the highest for OI and/or sepsis and the lowest for UTI.

The frequency and rate of sepsis increased over time. Sepsis surpassed pneumonia as the most common serious infection in SSc in 2013–14. By 2015–2016, sepsis was twice as common as pneumonia. This is an important observation. This trend in SSc cohort may reflect the increase in hospitalizations with sepsis in the general population [15]; systematic up-coding of severe infections to sepsis and misclassification error with sepsis diagnostic codes has been noted [16, 17]; and/or be related to the increased infection risk related to SSc and its treatments. An increase in the rate of hospitalized SSTI and OI in SSc over time may be due to increased recognition of these serious infections over time, a lower threshold for hospitalization for serious infections, a higher rate of use of immunosuppressive drugs and/or glucocorticoids which increases the risk of serious infections, or an earlier recognition and more aggressive screening and treatment of cardio-pulmonary disease in SSc resulting in fewer hospitalizations for cardio-pulmonary disease and improving survival (and more hospitalizations for serious infections).

We noted a reduction in the in-hospital mortality from 10.3% in 1998–2000 to 7.8% in 2015–2016 for composite serious infection in people with SSc. Not surprisingly, the largest reductions were noted for the serious infections with the highest mortality in 1998–2000. We noted large reductions of in-hospital mortality for OI, 21.2 to 7.7%; sepsis, 22.5 to 13.2%; and pneumonia, 9.8 to 2.8%, from 1998–2000 to 2015–2016, respectively. To our knowledge, these are important novel findings in a SSc cohort hospitalized with serious infections. Survival rates in SSc have improved over time [18], which our findings further validate. As expected, in-hospital mortality of 9% in people with serious infections and SSc in our study is slightly higher than the reported overall in-hospital mortality rates in SSc of 7.1% using the 1995 NIS [10] and 6.3% using the 2002–2003 NIS [3]. Higher unadjusted in-hospital mortality in SSc patients (9%) versus the general population (6.2%) hospitalized with serious infections in our study is consistent with the finding of similar or higher mortality in the intensive care unit admission in people with rheumatic diseases versus not [19].

We noted a 3.6-fold increase in the mean hospital charges with a 9% concomitant decrease in the mean/median hospital stay for serious infections in SSc patients, from 1998–2000 to 2015–2016. These trends over time are consistent with the general trends in the overall NIS cohort. The reduction in median hospital stay was the greatest for OI, from 9.5 to 5.8 days, and minimal for sepsis, from 5.5 to 5.3 days. In 2015–2016, mean hospital charges for SSTI were similar to that of pneumonia in people with SSc, which may be partially related to associated Raynaud’s disease and digital ulcers.

Multivariable-adjusted analyses showed lower odds for healthcare utilization and in-hospital mortality for all serious infections compared to sepsis. The reduction in odds was 28–62% for healthcare utilization and 54–98% for in-hospital mortality. These important differences separate sepsis from other serious infections in SSc, with regard to outcomes.

Several other factors were also independently associated with poorer healthcare utilization and in-hospital mortality outcomes. A higher Deyo-Charlson score ≥ 2 was associated with higher healthcare utilization and in-hospital mortality, and odds were increased by 37–54%. Our finding validates findings from another study that showed that diabetes, anxiety, and depression increased in-hospital mortality in hospitalized SSc patients [20], and extends it to SSc patients hospitalized with serious infections.

Our finding of an independent association of unmodifiable factors such as older age, Medicare or Medicaid insurance payer, Northeast location, urban teaching or non-teaching status, and medium or large hospital bed size with higher odds of healthcare utilization can help in a better understanding of associated healthcare utilization.

Our study has several limitations. Misclassification bias is possible, since we used the ICD-9-CM or ICD-10-CM codes to identify people with SSc and infections. However, this bias may be minimal since the diagnostic codes for SSc [10] and serious infections [7–9] have been shown to be valid in previous studies. Since the NIS counts discharges, longitudinal outcome analyses were not possible at a patient-level, including the 30- and 90-day post-discharge readmission and mortality risk. NIS does not provide data on disease characteristics (duration, severity, end-organ involvement, complications), current medications (immunosuppressives, glucocorticoids), details of medication use during the index hospitalization, and laboratory or imaging study results. Therefore, we were unable to assess the association of these disease and treatment variables with the risk of serious infections or associated time trends and outcomes. Future studies need to address these important questions. Hospital charges were not adjusted for inflation over 2 decades, and some trends noted in charges are due to inflation; however, a 2–3-fold increase in mean/median hospital charges cannot all be attributed to inflation. NIS provides charges, not actual costs, which may be lower than the charges. The NIS does not include data from the military or Veterans Affairs hospitals, which can lead to some selection bias.

The strengths of our study are the use of national US data that can produce national estimates of mortality, charges and healthcare utilization, the inclusion of several covariates and confounders in regression analyses resulting in robust estimates of association, and inclusion of a large sample size.

## Conclusions

In conclusion, we found important differences in hospitalized serious infection patients between SSc vs. non-SSc. In the USA, the most common serious infection in hospitalized SSc patients was pneumonia followed by sepsis; sepsis was the most common in the most recent study period. We noted a significant increase in the rate of sepsis, and possibly OI and UTI. Hospital charges increased, and the duration of hospital stay and in-hospital mortality decreased from 1998–2000 to 2015–2016. We identified several modifiable and non-modifiable independent risk factors for poorer outcomes that can help policymakers and spark new interventions for improving outcomes.

## Supplementary information

**Additional file 1 **: **Appendix 1**. ICD-10-CM codes for serious infections. **Appendix 2**. Characteristics of people with serious infection in cohorts with versus without systemic sclerosis. **Appendix 3**. Characteristics of patients with each hospitalized serious infection as primary diagnosis in people with systemic sclerosis as a secondary diagnosis. **Appendix 4**. Frequency of serious infections in people with systemic sclerosis over time. **Appendix 5**. Serious Infection Rate in the general NIS cohort per 100,000 NIS population. **Appendix 6**. Rate of serious infections in people with systemic sclerosis over time using two denominators. **Appendix 7**. Contrast between the first and the last study periods, 1998-2000 versus 2015-2016 for healthcare utilization and mortality outcomes in people with systemic sclerosis. **Appendix 8**. Time-trends in the length of hospital stay and total hospital charges across all study periods, 1998-2000 from 2015-2016 in people with systemic sclerosis hospitalized with each serious infection

## Data Availability

These data are easily available from the Agency for Healthcare Research and Quality (AHRQ’s) “Healthcare Cost and Utilization Project (HCUP)” and can be obtained after completing an online Data Use Agreement training session and signing a Data Use Agreement. The contact information for requesting the data is as follows: HCUP Central Distributor Phone: (866) 556-4287 (toll-free) Fax: (866) 792-5313 E-mail: HCUPDistributor@ahrq.gov

## Abbreviations

NIS: National Inpatient Sample
HR: Hazard ratio
CI: Confidence interval
SD: Standard deviation
UTI: Urinary tract infection
SSTI: Skin and soft tissue infections
OI: Opportunistic infections
CCS: Clinical Classifications Software
UAB: University of Alabama at Birmingham
ICD-9-CM: International Classification of Diseases, Ninth Revision, Clinical Modification
