# Supplementary material for: Serious infections in people with systemic sclerosis: a national US study
Source: Arthritis Res Ther. 2020 Jun 29;22:163. doi: 10.1186/s13075-020-02216-w (PMC7322895; doi:10.1186/s13075-020-02216-w)
Supplement: Supplementary file 1 — Additional file 1 : Appendix 1. ICD-10-CM codes for serious infections. Appendix 2. Characteristics of people with serious infection in cohorts with versus without systemic sclerosis. Appendix 3. Characteristics of patients with each hospitalized serious infection as primary diagnosis in people with systemic sclerosis as a secondary diagnosis. Appendix 4. Frequency of serious infections in people with systemic sclerosis over time. Appendix 5. Serious Infection Rate in the general NIS cohort per 100,000 NIS population. Appendix 6. Rate of serious infections in people with systemic sclerosis over time using two denominators. Appendix 7. Contrast between the first and the last study periods, 1998-2000 versus 2015-2016 for healthcare utilization and mortality outcomes in people with systemic sclerosis. Appendix 8. Time-trends in the length of hospital stay and total hospital charges across all study periods, 1998-2000 from 2015-2016 in people with systemic sclerosis hospitalized with each serious infection [file 13075_2020_2216_MOESM1_ESM.docx]

**Appendix**

**Appendix 1. ICD-10-CM codes for serious infections**

| Opportunistic infections (OI) | 'B25.x', 'B02.xx', 'B37.xx', 'B58.xx', 'B59', 'B45.x', 'A32.xx', 'A43.x', 'B44.xx', 'B38.xx', 'B39.x', 'B40.xx', 'B27.1x', 'A15.7', 'A15.6', 'A15.0', 'A15.5', 'A15.6', 'A15.4', 'A15.8', 'A17.0', 'A17.1', 'A17.9', 'A18.4', 'A18.2', 'A18.6', 'A18.7', ’A19.2', 'A19.8', 'A19.9', 'B27.0x', 'A31.0', 'A31.1', 'A31.2', 'A31.8', 'A31.9', 'A17.81', 'A17.82', 'A17.89', 'A18.31', 'A18.32', 'A18.39', 'A18.01', 'A18.02', 'A18.03', 'A18.11', 'A18.12', 'A18.13', 'A18.15', 'A18.14', 'A18.17', 'A18.16', 'A18.18', 'A18.10', 'A18.50', 'A18.51', 'A18.52', 'A18.53', 'A18.54', 'A18.59', 'A18.81', 'A18.85', 'A18.89', 'A18.84' |
| --- | --- |
| Skin and soft tissue infections (SSTI) | 'A46', 'B35.x', 'B36.x', 'L02', 'L03', 'B08.xxx', 'B09.xxx', 'A60.xx', 'L00', 'L01', 'L04', 'L05', 'L08', 'B00.1', 'B00.2', 'B00.7', 'B00.9', 'B01.8x', 'B01.9', 'B02.3x', 'B02.4', 'B02.5', 'B02.6', 'B02.7', 'B02.8', 'B02.9', 'B05.3', 'B05.4', 'B05.5', 'B05.6', 'B05.7', 'B05.8x', 'B05.9', 'B06.8x', 'B06.9', 'A36.3', 'K113', 'K114', 'K115', 'K116', 'K117', 'K118', 'K119', 'K120', 'K121', 'K122', 'L303', 'M726' |
| Urinary tract infection (UTI) | 'N10', 'N72', 'A54.xx', 'A55', 'A56.xx', 'N45', 'N410', 'N412', 'N413', 'N300', 'N700', 'N710', 'N390', 'N733', 'N771' |
| Pneumonia | 'J12', 'J13', 'J14', 'J15', 'J16', 'J17', 'J18', 'J10', 'J11', 'A48.1', 'B01.2', 'B05.2', 'B25.0' |
| Sepsis | 'A40.x', 'A41.xx', 'A32.7', 'R572', 'R650', 'R651' |

**Appendix 2. Characteristics of people with serious infection in cohorts with versus without systemic sclerosis**

|  | All hospitalization claims with a non-primary Systemic sclerosis diagnosis  (n= 478,319) | Hospitalized infection in people without Systemic sclerosis  (n=49,904,955) | Hospitalized infection in people with Systemic sclerosis  (n=61,615) |
| --- | --- | --- | --- |
| **Age, Mean (SE); Median** | 61.5 (0.08); 62.1 | 59.8 (0.08); 65.0 | 61.4 (0.15); 61.7 |
| **Age category** |  |  |  |
| <50 years | 94,673 (20.84%) | 14,069,367 (28.41%) | 13,304 (21.66%) |
| 50 - <65 years | 151,691 (33.39%) | 9,975,198 (20.14%) | 20,813 (33.88%) |
| 65 - 79 years | 158,140 (34.81%) | 13,267,620 (26.79%) | 20,364 (33.15%) |
| ≥80 years | 49,739 (10.95%) | 12,218,686 (24.67%) | 6,948 (11.31%) |
| **Sex** |  |  |  |
| Male | 69,282 (15.25%) | 23,449,357 (47.37%) | 9,834 (16.01%) |
| Female | 384,898 (84.75%) | 26,055,087 (52.63%) | 51,601 (83.99%) |
| **Race** |  |  |  |
| White | 273,140 (60.13%) | 29,722,254 (59.99%) | 36,617 (59.59%) |
| Black | 54,835 (12.07%) | 5,335,803 (10.77%) | 7,275 (11.84%) |
| Hispanic | 35,376 (7.79%) | 4,215,778 (8.51%) | 6,098 (9.92%) |
| Other/Missing | 90,923 (20.01%) | 10,274,741 (20.74%) | 11,454 (18.64%) |
| **Deyo-Charlson Index^1^ Score** |  |  |  |
| 0 | 0 (0%) | 15,683,828 (31.65%) | 0 (0%) |
| 1 | 151,810 (33.42%) | 12,915,197 (26.06%) | 22,179 (36.10%) |
| ≥2 | 302,469 (66.58%) | 20,954,335 (42.29%) | 39,266 (63.90%) |
| **Income Category** |  |  |  |
| 0-25^th^ percentile | 93,602 (21.03%) | 12,965,763 (26.80%) | 13,914 (23.13%) |
| 25-50^th^ percentile | 110,597 (24.85%) | 13,290,261 (27.47%) | 15,047 (25.01%) |
| 50-75^th^ percentile | 113,958 (25.61%) | 11,602,568 (23.98%) | 15,000 (24.93%) |
| 75-100^th^ percentile | 126,868 (28.51%) | 10,518,900 (21.74%) | 16,206 (26.93%) |
| **Insurance** |  |  |  |
| Private | 127,345 (28.08%) | 10,940,543 (22.13%) | 16,348 (26.65%) |
| Medicare | 268,822 (59.27%) | 27,438,957 (55.49%) | 36,620 (59.70%) |
| Medicaid | 40,457 (8.92%) | 7,081,259 (14.32%) | 6,184 (10.08%) |
| Other | 9,147 (2.02%) | 1,501,315 (3.04%) | 1,196 (1.95%) |
| Self | 7,802 (1.72%) | 2,484,821 (5.03%) | 995 (1.62%) |
| **Hospital Location/Teaching** |  |  |  |
| Rural | 48,537 (10.72%) | 7,025,707 (14.93%) | 6,989 (11.69%) |
| Urban | 163,400 (36.08%) | 19,233,904 (40.88%) | 22,105 (36.98%) |
| Urban Teaching | 240,910 (53.20%) | 20,791,119 (44.19%) | 30,687 (51.33%) |
|  |  |  |  |
| **Discharge Status** |  |  |  |
| Rehabilitation or skilled nursing facility (SNF) | 84,801 (19.78%) | 11,655,682 (25.42%) | 11,875 (21.40%) |
| Home | 344,000 (80.22%) | 34,201,298 (74.58%) | 43,619 (78.60%) |
| **Length of Stay in days** |  |  |  |
| ≤3 | 194,461 (42.81%) | 20,101,126 (40.56%) | 20,603 (33.53%) |
| >3 | 259,818 (57.19%) | 29,452,234 (59.44%) | 40,841 (66.47%) |
| **Died during hospitalization** | 22,276 (4.91%) | 3,073,566 (6.21%) | 5,503 (8.96%) |
| **Length of Stay in days: Mean (SE); median** | 6.1 (0.03); 3.6 | 6.0 (0.01); 3.7 | 6.7 (0.07); 4.4 |
| **Total hospital charges (US $)** |  |  |  |
| ≤median | 147,058 (32.37%) | 21,134,756 (42.65%) | 20,565 (33.47%) |
| >median | 307,221 (67.63%) | 28,418,604 (57.35%) | 40,879 (66.53%) |
| **Total hospital charges^2^ in US $: mean (SE); median** | 39,101 (458); 20,880 | 34,615 (166); 16,832 | 44,121 (875); 22,105 |
| 1998-2000 | 18,422 (491); 9,967 | 18,063 (789); 9,983 | 18,264 (339); 9,619 |
| 2015-2016 | 60,106 (1,395); 34,562 | 53,550 (430); 28,770 | 64,361 (2,414); 36,369 |
| SE, standard error; $, dollar  ^1^ Deyo-Charlson index consists of 17 comorbidities: Myocardial Infarction; Congestive heart disease; peripheral vascular disease; cerebrovascular disease; dementia; chronic pulmonary disease; rheumatologic disease; peptic ulcer disease; mild liver disease; diabetes; diabetes with complications; hemiplegia or paraplegia; renal disease; any malignancy, including leukemia and lymphoma; moderate or severe liver disease; metastatic solid tumor; AIDS  ^2^ Median total charges by year: 1998, $5,775; 1999, $6,060; 2000, $6,723; 2001, $7,504; 2002, $8,601; 2003, $9,732; 2004, $9918; 2005, $10,816; 2006, $12,078; 2007, $13,001; 2008, $13,983; 2009, $14,814; 2010, $15,560; 2011, $17,815; 2012, $19,654; 2013, $21,166; 2014, $22,343; 2015, $23,678; 2016, $25,261 | | | |

**Appendix 3. Characteristics of patients with each hospitalized serious infection as primary diagnosis in people with systemic sclerosis as a secondary diagnosis**

|  | OI  (n=1,531; 2.5%) | SSTI  (n=11,423; 18.5%) | UTI  (n=1,654; 2.7%) | Pneumonia  (n=27,471; 44.6%) | Sepsis  (n=19,536; 31.7%) | Composite Infection  (n=61,615) |
| --- | --- | --- | --- | --- | --- | --- |
| **Age, Mean (SE); Median** | 56.8 (0.92); 56.4 | 58.6 (0.35); 58.5 | 58.6 (0.94); 59.1 | 62.4 (0.21); 63.1 | 62.1 (0.24); 62.1 | 61.4 (0.15); 61.7 |
| **Age category** |  |  |  |  |  |  |
| <50 years | 503 (32.8%) | 3,106 (27.3%) | 532 (32.2%) | 5,357 (19.6%) | 3,806 (19.5%) | 13,304 (21.7%) |
| 50 - <65 years | 470 (30.7%) | 4,092 (35.9%) | 473 (28.7%) | 8,952 (32.8%) | 6,826 (35.0%) | 20,813 (33.9%) |
| 65 - 79 years | 452 (29.5%) | 3,128 (27.5%) | 425 (25.8%) | 9,662 (35.4%) | 6,696 (34.3%) | 20,364 (33.1%) |
| ≥80 years | 106 (6.9%) | 1,065 (9.4%) | 219 (13.3%) | 3,360 (12.3%) | 2,197 (11.3%) | 6,948 (11.3%) |
| **Sex** |  |  |  |  |  |  |
| Male | 224 (14.7%) | 1,814 (15.9%) | 84 (5.1%) | 4,560 (16.7%) | 3,152 (16.1%) | 9,834 (16.0%) |
| Female | 1,306 (85.3%) | 9,582 (84.1%) | 1,566 (94.9%) | 22,773 (83.3%) | 16,374 (83.9%) | 51,601 (84.0%) |
| **Race** |  |  |  |  |  |  |
| White | 855 (55.8%) | 7,073 (62.0%) | 1,052 (63.7%) | 15,930 (58.3%) | 11,708 (60.0%) | 36,617 (59.6%) |
| Black | 170 (11.1%) | 1,153 (10.1%) | 169 (10.3%) | 3,244 (11.9%) | 2,539 (13.0%) | 7,275 (11.8%) |
| Hispanic | 179 (11.7%) | 1,082 (9.5%) | 171 (10.4%) | 2,540 (9.3%) | 2,126 (10.9%) | 6,098 (9.9%) |
| Other/Missing | 327 (21.4%) | 2,092 (18.4%) | 258 (15.6%) | 5,623 (20.6%) | 3,153 (16.1%) | 11,454 (18.6%) |
| **Deyo-Charlson Score** |  |  |  |  |  |  |
| 0 | 0 (0%) | 0 (0%) | 0 (0%) | 0 (0%) | 0 (0%) | 0 (0%) |
| 1 | 658 (43.0%) | 5,427 (47.6%) | 763 (46.2%) | 9,528 (34.9%) | 5,803 (29.7%) | 22,179 (36.1%) |
| ≥2 | 873 (57.0%) | 5,973 (52.4%) | 887 (53.8%) | 17,809 (65.1%) | 13,723 (70.3%) | 39,266 (63.9%) |
| **Income Category** |  |  |  |  |  |  |
| 0-25^th^ percentile | 375 (25.1%) | 2,549 (23.0%) | 353 (22.0%) | 5,986 (22.4%) | 4,652 (24.2%) | 13,914 (23.1%) |
| 25-50^th^ percentile | 272 (18.2%) | 2,723 (24.5%) | 493 (30.8%) | 7,026 (26.2%) | 4,534 (23.6%) | 15,047 (25.0%) |
| 50-75^th^ percentile | 394 (26.4%) | 2,713 (24.4%) | 350 (21.8%) | 6,503 (24.3%) | 5,040 (26.3%) | 15,000 (24.9%) |
| 75-100^th^ percentile | 451 (30.2%) | 3,121 (28.1%) | 406 (25.4%) | 7,260 (27.1%) | 4,968 (25.9%) | 16,206 (26.9%) |
| **Insurance** |  |  |  |  |  |  |
| Private | 497 (32.5%) | 3,429 (30.1%) | 463 (28.2%) | 7,268 (26.6%) | 4,691 (24.1%) | 16,348 (26.6%) |
| Medicare | 782 (51.1%) | 6,077 (53.3%) | 913 (55.7%) | 16,707 (61.2%) | 12,141 (62.3%) | 36,620 (59.7%) |
| Medicaid | 194 (12.7%) | 1,382 (12.1%) | 175 (10.7%) | 2,387 (8.7%) | 2,046 (10.5%) | 6,184 (10.1%) |
| Other | 48 (3.1%) | 255 (2.2%) | 34 (2.1%) | 511 (1.9%) | 348 (1.8%) | 1,196 (1.9%) |
| Self | 9 (0.6%) | 252 (2.2%) | 54 (3.3%) | 417 (1.5%) | 262 (1.3%) | 995 (1.6%) |
| **Hospital Region** |  |  |  |  |  |  |
| Northeast | 242 (15.8%) | 2,801 (24.5%) | 286 (17.3%) | 5,034 (18.3%) | 3,363 (17.2%) | 11,726 (19.0%) |
| Midwest | 396 (25.9%) | 2,544 (22.3%) | 337 (20.4%) | 6,600 (24.0%) | 4,216 (21.6%) | 14,093 (22.9%) |
| South | 532 (34.8%) | 3,860 (33.8%) | 683 (41.3%) | 10,566 (38.5%) | 7,032 (36.0%) | 22,674 (36.8%) |
| West | 361 (23.6%) | 2,218 (19.4%) | 348 (21.1%) | 5,271 (19.2%) | 4,925 (25.2%) | 13,122 (21.3%) |
| **Hospital Location/Teaching** |  |  |  |  |  |  |
| Rural | 99 (6.6%) | 1,332 (12.0%) | 197 (12.5%) | 3,728 (14.1%) | 1,633 (8.5%) | 6,989 (11.7%) |
| Urban | 457 (30.4%) | 3,721 (33.5%) | 540 (34.2%) | 10,501 (39.8%) | 6,885 (35.9%) | 22,105 (37.0%) |
| Urban Teaching | 948 (63.1%) | 6,048 (54.5%) | 842 (53.3%) | 12,167 (46.1%) | 10,683 (55.6%) | 30,687 (51.3%) |
| **Hospital Bed size** |  |  |  |  |  |  |
| Small | 126 (8.3%) | 1,641 (14.4%) | 250 (15.1%) | 4,346 (15.9%) | 2,532 (13.0%) | 8,895 (14.5%) |
| Medium | 316 (20.7%) | 2,875 (25.2%) | 457 (27.7%) | 6,940 (25.3%) | 5,091 (26.2%) | 15,679 (25.5%) |
| Large | 1,084 (71.0%) | 6,883 (60.4%) | 942 (57.2%) | 16,102 (58.8%) | 11,833 (60.8%) | 15,679 (25.5%) |
|  |  |  |  |  |  |  |
| **Discharge Status** |  |  |  |  |  |  |
| Rehabilitation or nursing facility | 202 (14.4%) | 1,600 (14.4%) | 239 (14.7%) | 4,561 (17.9%) | 5,272 (33.2%) | 11,875 (21.4%) |
| Home | 1,202 (85.6%) | 9,520 (85.6%) | 1,395 (85.3%) | 20,903 (82.1%) | 10,599 (66.8%) | 43,619 (78.6%) |
| **Length of Stay in days** |  |  |  |  |  |  |
| ≤3 | 320 (20.9%) | 4,402 (38.6%) | 911 (55.2%) | 9,582 (35.1%) | 5,387 (27.6%) | 20,603 (33.5%) |
| >3 | 1,210 (79.1%) | 6,998 (61.4%) | 739 (44.8%) | 17,755 (64.9%) | 14,139 (72.4%) | 40,841 (66.5%) |
| **Died during hospitalization** | 126 (8.2%) | 147 (1.3%) | 5 (0.3%) | 1,672 (6.1%) | 3,553 (18.2%) | 5,503 (9.0%) |
| **Length of Stay in days: Mean (SE); median** | 9.4 (0.57); 6.1 | 5.5 (0.11); 3.7 | 4.1 (0.15); 2.8 | 6.2 (0.09); 4.1 | 8.2 (0.14); 5.4 | 6.7 (0.07); 4.4 |
| **Total hospital charges (US $)** |  |  |  |  |  |  |
| ≤median | 395 (25.8%) | 5,069 (44.5%) | 896 (54.3%) | 9,556 (35.0%) | 4,649 (23.8%) | 20,565 (33.5%) |
| >median | 1,136 (74.2%) | 6,332 (55.5%) | 754 (45.7%) | 17,781 (65.0%) | 14,877 (76.2%) | 40,879 (66.5%) |
| **Total Charge in US $: mean (SE); median** | 65,855 (11,804); 31,692 | 25,150 (787); 15,418 | 21,205 (1,245); 13,646 | 32,908 (795); 18,536 | 71,874 (2,051); 38,118 | 44,121 (875); 22,105 |

**Appendix 4.** **Frequency of serious infections in people with systemic sclerosis over time**

|  | OI | SSTI | UTI | Pneumonia | Sepsis | Composite Infection |
| --- | --- | --- | --- | --- | --- | --- |
| 1998-2000 | 165 | 1,297 | 155 | 3,838 | 1,104 | 6,558 |
| 2001-2002 | 125 | 933 | 142 | 2,630 | 748 | 4,579 |
| 2003-2004 | 182 | 1,070 | 144 | 2,962 | 878 | 5,237 |
| 2005-2006 | 168 | 1,312 | 145 | 3,120 | 1,375 | 6,120 |
| 2007-2008 | 166 | 1,225 | 176 | 2,936 | 1,469 | 5,972 |
| 2009-2010 | 152 | 1,339 | 130 | 3,061 | 1,984 | 6,666 |
| 2011-2012 | 212 | 1,511 | 147 | 3,108 | 3,004 | 7,982 |
| 2013-2014 | 165 | 1,445 | 125 | 2,965 | 3,935 | 8,635 |
| 2015-2016* | 195 | 1,290 | 490 | 2,850 | 5,040 | 9,865 |
| The ICD-9 codes used to define each infection category were as follows:  opportunistic infections (OIs) that included tuberculosis (010–018), nontuberculous mycobacteria (031), cytomegalovirus (078.5), Epstein-Barr virus (075), herpes zoster (053), candidiasis (112.4, 112.5, 112.81, 112.83), toxoplasmosis (130), pneumocystosis (136.3), cryptococcosis (117.5), listeriosis (027.0), nocardiosis (039), aspergillosis (117.3), coccidioidomycosis (114), histoplasmosis (115), and blastomycosis (116.0);  skin and soft tissue infections (SSTIs; 040.0, 569.61, 681, 682, 785.4, 728.86, and 035);  urinary tract infection (UTI; 590);  pneumonia (003.22, 481.0, 513.0, 480, 482, 483, 485, and 486);  sepsis/bacteremia (hereafter, sepsis; 038 and 790.7);  *Estimates in this period reflect the only study period where ICD-codes transitioned from ICD-9-CM to ICD-10-CM, and therefore may be a little unstable | | | | | | |

**Appendix 5. Serious Infection Rate in the general NIS cohort per 100,000 NIS population**

|  | OI | SSTI | UTI | Pneumonia | Sepsis | Composite Infection | Total claims |
| --- | --- | --- | --- | --- | --- | --- | --- |
| 1998-2000 | 159.53 | 974.93 | 313.48 | 3,398.42 | 989.47 | 5,835.83 | 103,665,051 |
| 2001-2002 | 139.43 | 1,097.63 | 335.19 | 3,303.16 | 919.34 | 5,794.75 | 72,617,381 |
| 2003-2004 | 138.89 | 1,269.23 | 352.31 | 3,260.20 | 1,088.62 | 6,109.25 | 74,571,583 |
| 2005-2006 | 143.86 | 1,490.70 | 350.89 | 3,235.99 | 1,451.26 | 6,672.71 | 75,919,595 |
| 2007-2008 | 138.64 | 1,531.68 | 336.98 | 2,891.86 | 1,834.90 | 6,734.07 | 76,366,797 |
| 2009-2010 | 138.62 | 1,627.18 | 350.97 | 2,849.14 | 2,223.62 | 7,189.53 | 75,086,597 |
| 2011-2012 | 130.48 | 1,688.87 | 343.20 | 2,791.79 | 2,948.98 | 7,903.32 | 73,447,261 |
| 2013-2014 | 121.39 | 1,665.59 | 324.30 | 2,578.24 | 3,939.63 | 8,629.14 | 70,956,610 |
| 2015-2016* | 110.92 | 1,667.92 | 882.11 | 2,401.02 | 5,109.07 | 10,171.05 | 71,445,363 |
| *Estimates in this period reflect the only study period where ICD-codes transitioned from ICD-9-CM to ICD-10-CM, and therefore may be a little unstable | | | | | | | |

**Appendix 6.** **Rate of serious infections in people with systemic sclerosis over time using two denominators**

|  | OI | SSTI | UTI | Pneumonia | Sepsis | Composite Infection | Total NIS claims |
| --- | --- | --- | --- | --- | --- | --- | --- |
|  | **Rate per 100,000 NIS claims** | | | | | | |
| 1998-2000 | 0.16 | 1.25 | 0.15 | 3.70 | 1.06 | 6.33 | 103,665,051 |
| 2001-2002 | 0.17 | 1.28 | 0.20 | 3.62 | 1.03 | 6.31 | 72,617,381 |
| 2003-2004 | 0.24 | 1.43 | 0.19 | 3.97 | 1.18 | 7.02 | 74,571,583 |
| 2005-2006 | 0.22 | 1.73 | 0.19 | 4.11 | 1.81 | 8.06 | 75,919,595 |
| 2007-2008 | 0.22 | 1.60 | 0.23 | 3.84 | 1.92 | 7.82 | 76,366,797 |
| 2009-2010 | 0.20 | 1.78 | 0.17 | 4.08 | 2.64 | 8.88 | 75,086,597 |
| 2011-2012 | 0.29 | 2.06 | 0.20 | 4.23 | 4.09 | 10.87 | 73,447,261 |
| 2013-2014 | 0.23 | 2.04 | 0.18 | 4.18 | 5.55 | 12.17 | 70,956,610 |
| 2015-2016 | 0.27 | 1.81 | 0.69 | 3.99 | 7.05 | 13.81 | 71,445,363 |
| P-value* | <0.0001 | <0.0001 | <0.0001 | <0.0001 | <0.0001 | <0.0001 |  |
|  | **Rate per 100,000 systemic sclerosis** **claims** | | | | | | |
|  | OI | SSTI | UTI | Pneumonia | Sepsis | Composite Infection | Systemic sclerosis claims |
| 1998-2000 | 287.92 | 2263.25 | 270.47 | 6697.26 | 1926.47 | 11443.63 | 57,307 |
| 2001-2002 | 288.69 | 2154.78 | 327.95 | 6074.04 | 1727.52 | 10575.30 | 43,299 |
| 2003-2004 | 388.23 | 2282.42 | 307.17 | 6318.26 | 1872.87 | 11171.08 | 46,880 |
| 2005-2006 | 325.51 | 2542.04 | 280.94 | 6045.11 | 2664.11 | 11857.71 | 51,612 |
| 2007-2008 | 325.40 | 2401.30 | 345.00 | 5755.28 | 2879.60 | 11706.59 | 51,014 |
| 2009-2010 | 272.28 | 2398.61 | 232.87 | 5483.30 | 3554.03 | 11941.10 | 55,824 |
| 2011-2012 | 361.50 | 2576.56 | 250.67 | 5299.77 | 5122.43 | 13610.94 | 58,644 |
| 2013-2014 | 292.99 | 2565.92 | 221.97 | 5265.03 | 6987.48 | 15333.39 | 56,315 |
| 2015-2016 | 339.57 | 2246.41 | 853.29 | 4963.00 | 8776.67 | 17178.93 | 57,425 |
| P-value* | <0.0001 | <0.0001 | <0.0001 | <0.0001 | <0.0001 | <0.0001 |  |
| *P-value is from Cochran-Armitage two sided test for trend  The first time-period is 3-year and the subsequent ones are 2-year duration  Composite infection indicates any of the five infections as the primary diagnosis | | | | | | | |

**Appendix 7. Contrast between the first and the last study periods, 1998-2000 versus 2015-2016 for healthcare utilization and mortality outcomes in people with systemic sclerosis**

|  | OI | SSTI | UTI | Pneumonia | Sepsis | Composite Infection |
| --- | --- | --- | --- | --- | --- | --- |
| **Length of Stay in days** >3 days |  |  |  |  |  |  |
| 1998-2000 | 160 (97.2%) | 864 (66.6%) | 65 (42.0%) | 2,764 (72.0%) | 818 (74.1%) | 4,671 (71.2%) |
| 2015-2016 | 155 (79.5%) | 680 (52.7%) | 215 (43.9%) | 1,680 (58.9%) | 3,625 (71.9%) | 6,355 (64.4%) |
| **Died during hospitalization** |  |  |  |  |  |  |
| 1998-2000 | 35 (21.2%) | 16 (1.2%) | 0 (0.0%) | 375 (9.8%) | 248 (22.5%) | 674 (10.3%) |
| 2015-2016 | 15 (7.7%) | <10 (N/A) | 0 (0.0%) | 80 (2.8%) | 665 (13.2%) | 765 (7.8%) |
| **Length of Stay in days: Mean (SE); median** |  |  |  |  |  |  |
| 1998-2000 | 12.5 (1.64); 9.5 | 6.5 (0.40); 4.2 | 3.7 (0.32); 2.7 | 6.9 (0.23); 4.6 | 8.9 (0.81); 5.5 | 7.2 (0.24); 4.6 |
| 2015-2016 | 9.8 (1.69); 5.8 | 4.9 (0.27); 3.2 | 3.9 (0.28); 2.7 | 5.0 (0.17); 3.6 | 8.0 (0.27); 5.3 | 6.6 (0.16); 4.2 |
| **Total hospital charges (US $)** >median |  |  |  |  |  |  |
| 1998-2000 | 160 (97.3%) | 822 (63.3%) | 82 (53.2%) | 2,657 (69.2%) | 840 (76.1%) | 4,560 (69.5%) |
| 2015-2016 | 140 (71.8%) | 635 (49.2%) | 210 (42.9%) | 1,580 (55.4%) | 3,735 (74.1%) | 6,300 (63.9%) |
| **Total Charge in US $: mean (SE); median** |  |  |  |  |  |  |
| 1998-2000 | 37,031 (6,824); 20,855 | 13,003 (1,114); 8,266 | 8,593 (904); 6,858 | 17,440 (1,030); 9,678 | 23,152 (2,037); 13,537 | 17,793 (769); 9,882 |
| 2015-2016 | 79,738 (16,861); 42,236 | 40,692 (3,805); 23,886 | 30,903 (2,923); 21,519 | 40,754 (2,018); 28,046 | 87,095 (4,244); 51,182 | 64,361 (2,414); 36,369 |
| In accordance with NIS instructions, cells with frequencies less than 10 can not be shown, and are being presented as <10 in this table. | | | | | | |

**Appendix 8**. **Time-trends in the length of hospital stay and total hospital charges across all study periods, 1998-2000 from 2015-2016 in people with systemic sclerosis hospitalized with each serious infection**

|  | OI | SSTI | UTI | Pneumonia | Sepsis | Composite Infection |
| --- | --- | --- | --- | --- | --- | --- |
| **Hospital Stay in days: Mean (SE); median** |  |  |  |  |  |  |
| 1998-2000 | 12.5 (1.64); 9.5 | 6.5 (0.40); 4.2 | 3.7 (0.32); 2.7 | 6.9 (0.23); 4.6 | 8.9 (0.81); 5.5 | 7.2 (0.24); 4.6 |
| 2001-2002 | 7.7 (1.32); 4.4 | 6.4 (0.41); 4.4 | 4.8 (0.68); 2.9 | 6.8 (0.48); 4.6 | 8.2 (0.58); 6.2 | 6.2 (0.09); 4.3 |
| 2003-2004 | 8.3 (1.22); 6.1 | 6.0 (0.36); 4.2 | 4.6 (0.59); 2.8 | 6.8 (0.32); 4.2 | 9.4 (0.64); 6.4 | 5.9 (0.07); 4.1 |
| 2005-2006 | 10.7 (2.42); 5.7 | 5.7 (0.42); 3.7 | 4.1 (0.36); 3.3 | 5.9 (0.2); 4.0 | 8.1 (0.46); 5.7 | 5.8 (0.06); 4.0 |
| 2007-2008 | 7.1 (0.87); 5.6 | 5.5 (0.27); 4.0 | 4.3 (0.47); 2.9 | 6.4 (0.26); 4.3 | 8.6 (0.46); 6.1 | 5.8 (0.06); 4.0 |
| 2009-2010 | 6.9 (0.98); 3.9 | 5.1 (0.22); 3.8 | 3.4 (0.44); 2.4 | 6.0 (0.25); 3.8 | 9.7 (0.63); 5.9 | 6.2 (0.06); 4.2 |
| 2011-2012 | 12.2 (2.15); 6.3* | 5.4 (0.29); 3.6 | 4.2 (0.55); 3.0 | 5.9 (0.21); 3.8 | 8.0 (0.32); 5.2 | 6.2 (0.05); 4.1 |
| 2013-2014 | 7.5 (0.98); 6.1 | 4.6 (0.20); 3.3 | 3.8 (0.54); 2.1 | 5.8 (0.27); 4.0 | 7.4 (0.27); 4.9 | 6.2 (0.04); 4.1 |
| 2015-2016 | 9.8 (1.69); 5.8 | 4.9 (0.27); 3.2 | 3.9 (0.28); 2.7 | 5.0 (0.17); 3.6 | 8.0 (0.27); 5.3 | 6.6 (0.16); 4.2 |
| **Total Hospital charge in US $: mean (SE); median** |  |  |  |  |  |  |
| 1998-2000 | 37,031 (6,824); 20,855 | 13,003 (1,114); 8,266 | 8,593 (904); 6,858 | 17,440 (1,030); 9,678 | 23,152 (2,037); 13,537 | 17,793 (769); 9,882 |
| 2001-2002 | 30,111 (9,007); 10,621 | 16,110 (1,595); 9,350 | 11,300 (1,888); 6,739 | 21,498 (2,174); 13,234 | 28,456 (2,096); 21,227 | 21,441 (1,371); 12,665 |
| 2003-2004 | 44,432 (9,874); 24,939 | 18,659 (1,922); 11,495 | 14,199 (2,389); 9,093 | 27,810 (2,002); 14,860 | 44,307 (4,151); 24,076 | 28,869 (1,493); 15,098 |
| 2005-2006 | 59,853 (17,267); 25,863 | 22,272 (3,301); 12,872 | 17,517 (2,771); 11,596 | 26,296 (1,633); 15,152 | 45,974 (3,214); 30,426 | 30,517 (1,477); 17,080 |
| 2007-2008 | 37,404 (4,491); 30,940 | 26,472 (2,064); 17,311 | 19,453 (2,594); 13,136 | 34,360 (2,482); 20,772 | 63,165 (5,131); 33,020 | 39,422 (2,078); 22,229 |
| 2009-2010 | 50,144 (8,293); 34,331 | 24,818 (1,556); 17,780 | 22,044 (5,956); 13,806 | 40,993 (2,994); 21,625 | 86,302 (7,733); 38,955 | 50,751 (3,247); 24,535 |
| 2011-2012 | 152,923 * (74,441); 52,535 | 31,507 (2,116); 20,308 | 20,242 (2,194); 17,130 | 41,137 (1,927); 26,917 | 78,834 (5,012); 42,837 | 55,766 (3,167); 29,416 |
| 2013-2014 | 64,352 (14,728); 32,399 | 27,785 (1,484); 19,869 | 24,112 (4,028); 16,090 | 47,956 (4,092); 28,902 | 81,049 (5,831); 40,194 | 59,257 (3,211); 32,022 |
| 2015-2016 | 79,738 (16,861); 42,236 | 40,692 (3,805); 23,886 | 30,903 (2,923); 21,519 | 40,754 (2,018); 28,046 | 87,095 (4,244); 51,182 | 64,361 (2,414); 36,369 |
| $, Dollar; SE, standard error; * estimates in 2011-2012 for OI had a larger SE and wider than usual range than other periods  Due to very few cases of in-hospital mortality, trends for each study period were not examined | | | | | | |
